# Thoracic aorta calcification but not inflammation is associated with increased cardiovascular disease risk: results of the CAMONA study

**DOI:** 10.1007/s00259-016-3552-9

**Published:** 2016-10-29

**Authors:** Björn A. Blomberg, Pim A. de Jong, Anders Thomassen, Marnix G. E. Lam, Werner Vach, Michael H. Olsen, Willem P. T. M. Mali, Jagat Narula, Abass Alavi, Poul F. Høilund-Carlsen

**Affiliations:** 1 Department of Nuclear Medicine, Odense University Hospital, Sdr. Boulevard 29, 5000 Odense C, Denmark; 2 Department of Radiology and Nuclear Medicine, University Medical Center Utrecht, Utrecht, The Netherlands; 3 Clinical Epidemiology, Institute of Medical Biometry and Medical Informatics, University Medical Center Freiburg, Freiburg, Germany; 4 The Cardiovascular and Metabolic Preventive Clinic, Department of Endocrinology, Center for Individualized Medicine in Arterial Diseases, Odense University Hospital, Odense, Denmark; 5 Icahn School of Medicine, Mount Sinai Hospital, New York, NY USA; 6 Department of Radiology, Hospital of the University of Pennsylvania, Philadelphia, PA USA; 7 Institute of Clinical Research, University of Southern Denmark, Odense, Denmark

**Keywords:** PET/CT, [^18^F]Fluorodeoxyglucose (^18^F-FDG), [^18^F]Sodium fluoride (Na^18^F), Arterial inflammation, Vascular calcification, Atherosclerosis

## Abstract

**Purpose:**

Arterial inflammation and vascular calcification are regarded as early prognostic markers of cardiovascular disease (CVD). In this study we investigated the relationship between CVD risk and arterial inflammation (^18^F-FDG PET/CT imaging), vascular calcification metabolism (Na^18^F PET/CT imaging), and vascular calcium burden (CT imaging) of the thoracic aorta in a population at low CVD risk.

**Methods:**

Study participants underwent blood pressure measurements, blood analyses, and ^18^F-FDG and Na^18^F PET/CT imaging. In addition, the 10-year risk for development of CVD, based on the Framingham risk score (FRS), was estimated. CVD risk was compared across quartiles of thoracic aorta ^18^F-FDG uptake, Na^18^F uptake, and calcium burden on CT.

**Results:**

A total of 139 subjects (52 % men, mean age 49 years, age range 21 – 75 years, median FRS 6 %) were evaluated. CVD risk was, on average, 3.7 times higher among subjects with thoracic aorta Na^18^F uptake in the highest quartile compared with those in the lowest quartile of the distribution (15.5 % vs. 4.2 %; *P* < 0.001). CVD risk was on average, 3.7 times higher among subjects with a thoracic aorta calcium burden on CT in the highest quartile compared with those in the lowest two quartiles of the distribution (18.0 % vs. 4.9 %; *P* < 0.001). CVD risk was similar in subjects in all quartiles of thoracic aorta ^18^F-FDG uptake.

**Conclusion:**

Our findings indicate that an unfavourable CVD risk profile is associated with marked increases in vascular calcification metabolism and vascular calcium burden of the thoracic aorta, but not with arterial inflammation.

**Electronic supplementary material:**

The online version of this article (doi:10.1007/s00259-016-3552-9) contains supplementary material, which is available to authorized users.

## Introduction

Adverse cardiovascular events and their sequelae are a major health concern in Western societies [1]. Efforts to prevent adverse cardiovascular events have focused on identifying asymptomatic individuals at high risk of cardiovascular disease (CVD), the so-called “vulnerable” patient [2, 3]. In theory, vulnerable patients benefit most from intensive evidence-based medical interventions. However, identifying the vulnerable patient remains a major ongoing challenge [2].

**Fig. 1:**
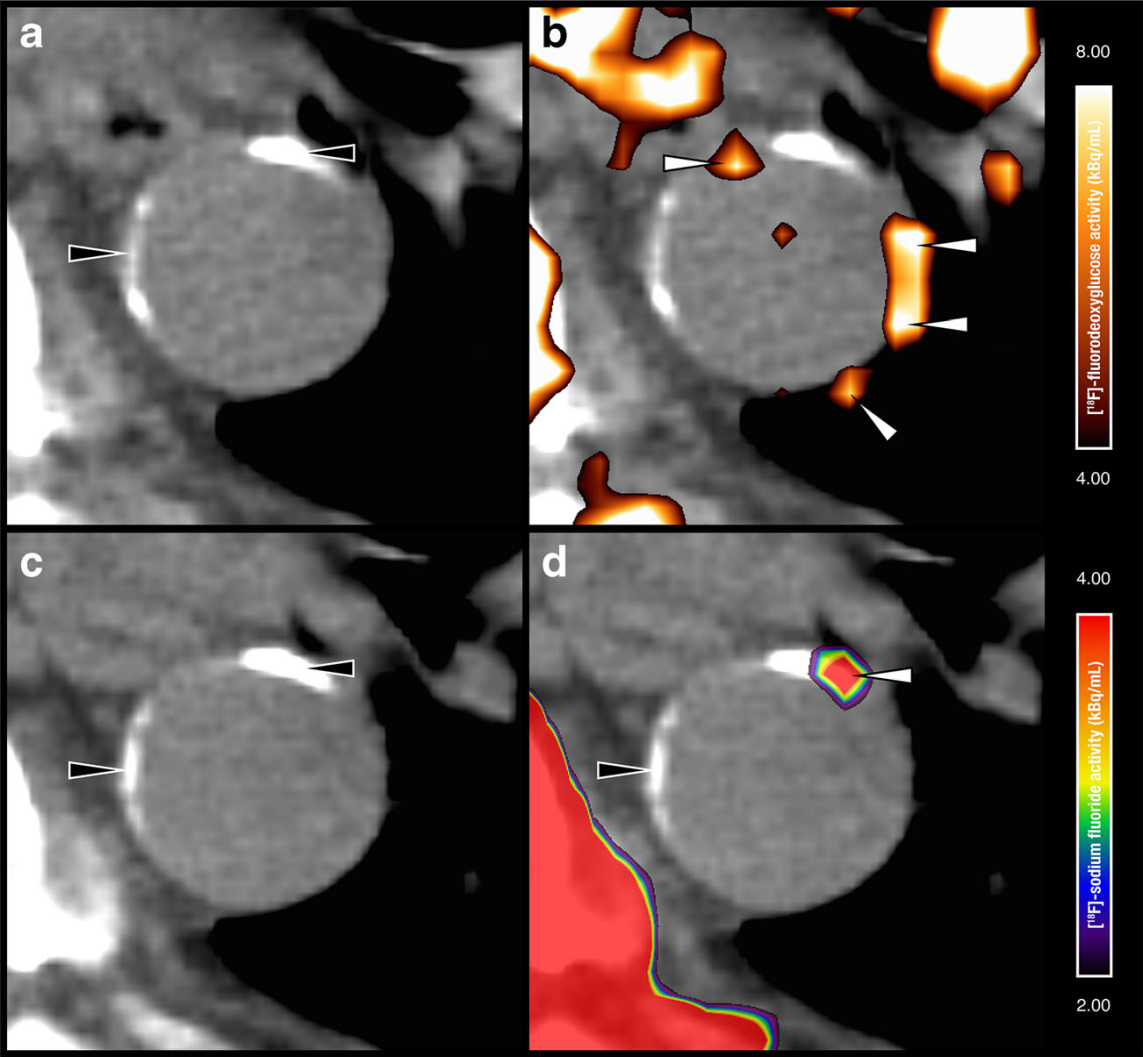
Axial CT (**a**, **c**), ^18^F-FDG PET/CT (**b**), and Na^18^F PET/CT (**d**) images obtained at the same location in 69-year-old man with hypertension, a body mass index of 28 kg/m^2^, and a Framingham risk score of 26 %. ^18^F-FDG accumulation is seen in the descending thoracic aorta (**b** *white arrowheads*), but not at sites with structural calcium deposits (**a**, **c** *black arrowheads*). In the Na^18^F PET/CT image (**d**) active (*white arrowhead*) and indolent (*black arrowhead*) vascular calcifications are distinguished

Recent developments in cardiovascular imaging, aimed at visualizing key pathophysiological processes in CVD, offer new opportunities for assessing patient vulnerability. Amongst others, arterial inflammation [4] and vascular calcification [5] have received attention as potent markers of increased CVD risk [6–8]. ^18^F-FDG PET/CT imaging can noninvasively assess arterial inflammation, whereas Na^18^F PET/CT and CT imaging can noninvasively assess vascular calcification (Fig. 1).

Although CVD risk in relation to arterial inflammation (^18^F-FDG PET/CT imaging), vascular calcification metabolism (Na^18^F PET/CT imaging), and vascular calcium burden (CT imaging) has been previously studied, in only a limited number of studies a combined approach has been applied [9, 10]. Moreover, few investigations have evaluated these cardiovascular imaging modalities in relation to CVD risk in a population at low CVD risk. Therefore, the aim of this study was to evaluate the relationship between CVD risk and arterial inflammation, vascular calcification metabolism, and vascular calcium burden in a cohort of subjects at low CVD risk, namely healthy volunteers and patients evaluated for chest pain syndromes.

## Materials and methods

This study was part of the “Cardiovascular Molecular Calcification Assessed by ^18^F-NaF PET/CT” (CAMONA) study. The CAMONA study was approved by the Danish National Committee on Health Research Ethics, registered at ClinicalTrials.gov (NCT01724749), and conducted in accordance with the principles of the Declaration of Helsinki. All study participants provided written informed consent.

### Subject selection

We recruited a heterogeneous population of subjects, including healthy volunteers and patients evaluated for chest pain syndromes. This allowed us to study the relationship between CVD risk and arterial inflammation, vascular calcification metabolism, and vascular calcium burden in a heterogeneous, but clinically relevant, group of subjects regarded to be at low CVD risk. Healthy volunteers were recruited from the general population by local advertisement or from the blood bank of Odense University Hospital, Denmark. Volunteers free of oncological disease, autoimmune disease, immunodeficiency syndromes, alcohol abuse, illicit drug use, (symptoms suggesting) CVD, or any prescription medication were considered healthy and were eligible for inclusion. Patients evaluated for chest pain syndromes were recruited from those referred for coronary CT angiography. Only patients with a 10-year risk of fatal CVD equal to or above 1 %, as estimated by the body mass index based the Systematic Coronary Risk Evaluation tool [11], were eligible for inclusion. Pregnant women were not considered for inclusion.

### Study design

Study participants were evaluated by questionnaires, blood pressure measurements, blood analyses, the Framingham risk score (FRS) [12], ^18^F-FDG PET/CT imaging, and Na^18^F PET/CT imaging. In addition, body weight and body mass index were determined. Questionnaires collected information about smoking habits, family history of CVD, and prescription medication. Blood pressure was measured three times after the subject had rested for of at least 30 min in the supine position. The average of the last two measurements was taken as the systolic and diastolic blood pressure. Blood analyses included fasting serum total cholesterol, serum LDL cholesterol, serum HDL cholesterol, serum triglycerides, fasting plasma glucose and glycated haemoglobin (HbA1c), and the Modification of Diet and Renal Disease (MDRD) equation was used to estimate glomerular filtration rate. In each subject, the 10-year risk of developing CVD was estimated using the FRS (i.e. risk of coronary death, myocardial infarction, coronary insufficiency, angina, ischaemic stroke, haemorrhagic stroke, transient ischaemic attack, peripheral artery disease, heart failure) based on age, gender, systolic blood pressure, total serum cholesterol, serum HDL cholesterol, smoking habit, and treatment for hypertension [12].

^18^F-FDG and Na^18^F PET/CT imaging were performed according to previously published methods [13, 14]. In summary, ^18^F-FDG and Na^18^F PET/CT imaging were performed on hybrid PET/CT systems (GE Discovery STE, VCT, RX, and 690/710 systems). Subjects were randomly allocated to a PET/CT system by the department’s booking system. PET/CT system specifications and image reconstruction parameters are summarized in Supplementary Table 1. ^18^F-FDG PET/CT imaging was performed 180 min after intravenous injection of 4.0 MBq of ^18^F-FDG per kilogram of body weight [13]. ^18^F-FDG was administered after an overnight fast of at least 8 h. Before ^18^F-FDG injection, the blood glucose concentration was determined to ensure a value below 8 mmol/L. On average, Na^18^F PET/CT imaging was performed within 2 weeks of ^18^F-FDG PET/CT imaging. Na^18^F PET/CT imaging was performed 90 min after intravenous injection of 2.2 MBq of Na^18^F per kilogram of body weight [14]. PET images were corrected for attenuation, scatter, random coincidences, and scanner dead time. Low–dose CT imaging (140 kV, 30 – 110 mA, noise index 25, 0.8 s per rotation, slice thickness 3.75 mm) was performed for attenuation correction, for anatomical orientation, and to determine the thoracic aorta CT calcium burden. The effective radiation dose received from the entire imaging protocol was approximately 14 mSv.

### Quantitative image analyses

All images were analysed using Philips IntelliSpace Portal Client, version 4.0. The image analyst was masked to the subject’s demographics and to the image specifications. Arterial ^18^F-FDG and Na^18^F uptake were quantified according to previously published methods [13–15]. In summary, uptake of ^18^F-FDG in the thoracic aorta was determined by manually placing an oval region of interest (ROI) around the outer perimeter of the artery on every slice of the axially oriented PET/CT images. For each ROI, the maximum decay-corrected ^18^F-FDG activity concentration was calculated. The maximum values obtained for each ROI were summed and divided by the number of ROIs, resulting in a single averaged maximum value (FDG_MAX_). Thoracic aorta NaF_MAX_ values were calculated in a similar manner. Blood ^18^F-FDG activity and blood Na^18^F activity were determined by drawing a single ROI in the lumen of the superior vena cava. Blood ^18^F-FDG activity and blood Na^18^F activity were quantified as the decay-corrected mean radiotracer activity concentration. Finally, the thoracic aorta CT calcium burden was calculated.

 The CT calcium burden was determined on low-dose CT images obtained as part of PET/CT imaging by calculating the calcium volume on every slice of the axially oriented CT images. Volumes obtained in each slice were summed and divided by the number of slices, resulting in a single mean CT calcium volume. The detection threshold for vascular calcium was set at 130 HU. The agreement between mean calcium volumes calculated on CT images from ^18^F-FDG PET/CT and those calculated from Na^18^F PET/CT was found to be excellent, and the CT images from ^18^F-FDG PET/CT were therefore used as reference for the statistical analysis. We could not assess interscan agreement for FDG_MAX_ and NaF_MAX_ because the ^18^F-FDG PET/CT and Na^18^F PET/CT scans were acquired only once.

### Statistical analysis

First, FDG_MAX_ and NaF_MAX_ were adjusted for blood activity, injected dose and PET/CT technology by multivariable linear regression, because previous studies had indicated that these parameters significantly affect the quantification of arterial ^18^F-FDG and Na^18^F uptake [15, 16]. Second, subject demographics are summarized by descriptive statistics and compared between healthy volunteers and patients using the unpaired Student’s *t* test, the Mann-Whitney *U* test, or Fisher’s exact test. Third, the correlations among thoracic aorta FDG_MAX_, thoracic aorta NaF_MAX_ and thoracic aorta CT calcium burden, were evaluated in terms of Spearman’s rank correlation coefficient (*ρ*). Fourth, the associations between cardiovascular risk factors and thoracic aorta FDG_MAX_, NaF_MAX_ and CT calcium burden were evaluated by univariate regression analysis, and those that were significantly associated were adjusted for age and sex. Linear models were extended by interaction terms to determine if the associations between cardiovascular risk factors and FDG_MAX_, NaF_MAX_ and CT calcium burden were modified by sex, subject recruitment (i.e. volunteers vs. patients), or prescription medication. Because no significant interactions were observed, results were not separated in relation to sex, volunteers/patients, or prescription medication. To establish independent determinants of thoracic aorta FDG_MAX_, NaF_MAX_, and CT calcium burden, variables selected on the basis of the results of the univariate analysis were entered into a multivariable linear regression analysis.

 The 10-year risks of CVD based on FRS were then estimated and these risk estimates were compared across quartiles of FDG_MAX_, NaF_MAX_ and CT calcium burden using factorial analysis of covariance (ANCOVA). The relationships between FRS and FDG_MAX_, NaF_MAX_ and CT calcium burden were also evaluated continuously using Spearman’s *ρ* and by multivariable linear regression analysis. All statistical analyses were repeated replacing FDG_MAX_ and NaF_MAX_ with the ratios of the maximum tracer activity in the aortic wall to the mean activity in the blood pool (target-to-background ratios, TBR) for ^18^F-FDG and Na^18^F (FDG-TBR_MAX/MEAN_ and NaF-TBR_MAX/MEAN_, respectively). TBR is commonly used to express aortic FDG and NaF uptake, but its use has been criticized [15, 16]. The results of these analyses are reported in Supplementary Fig. 2 and Supplementary Tables 5–7). Lastly, we assessed the agreement between the mean CT calcium volumes calculated on CT images from ^18^F-FDG PET/CT and those calculated on images from Na^18^F PET/CT in terms of the 95 % limits of agreement according to the method of Bland and Altman [17]. A two-tailed *P* value below 0.05 was regarded statistically significant. *P* values and 95 % confidence intervals were determined by a bootstrap of 2,000 samples. Statistical analyses were performed using SPSS Statistics, version 21 (IBM Corp., Armonk NY).

## Results

Between November 2012 and May 2014 89 healthy volunteers and 50 patients evaluated for chest pain syndromes were prospectively recruited. Several differences in subject demographics were observed between volunteers and patients (Table 1). These differences were mainly related to age, except for smoking habit, family history, HbA1c, FRS, and prescription medication, which remained significantly higher among patients compared with volunteers after adjustment for age. The ages of the study population ranged from 21 – 75 years and FRS ranged from 0.3 – 30.0 %.

**Table 1:** Subject demographics

	Volunteers (*n* =89)	Patients (*n* =50)	*P* value	Total (*N* =139)
Age (years), mean ± SD	44 ± 14	57 ± 11	<0.001*	49 ± 14
Male sex, *n* (%)	47 (53)	25 (50)	0.860	72 (52)
Smokers, *n* (%)				
Former	32 (36)	22 (44)	0.370	54 (39)
Current	3 (3)	10 (20)	0.002*	13 (9)
Family history, *n* (%)	16 (18)	19 (38)	0.014*	35 (25)
Blood pressure (mmHg), mean ± SD				
Systolic	128 ± 17	131 ± 17	0.277	129 ± 17
Diastolic	77 ± 10	79 ± 8	0.105	78 ± 10
Body mass index (kg/m^2^), mean ± SD	27 ± 4	27 ± 4	0.291	27 ± 4
Cholesterol (mmol/L), mean ± SD				
Total	4.9 ± 0.9	5.4 ± 0.9	0.006*	5.1 ± 0.9
LDL	3.1 ± 0.8	3.4 ± 0.9	0.037*	3.2 ± 0.8
HDL	1.4 ± 0.5	1.4 ± 0.4	0.834	1.4 ± 0.4
Triglycerides (mmol/L), mean ± SD	1.0 ± 0.7	1.2 ± 0.7	0.224	1.1 ± 0.7
Plasma glucose (mmol/L), mean ± SD	5.5 ± 0.5	5.9 ± 0.9	0.011*	5.6 ± 0.7
HbA1c (mmol/mol), mean ± SD	33.9 ± 4.1	37.4 ± 5.0	<0.001*	35.1 ± 4.7
eGFR (mL/min/1.73 m^2^), mean ± SD	82.9 ± 13.2	75.1 ± 14.3	0.002*	80.4 ± 14.1
Framingham risk score (%), median (25th, 75th percentile)	4 (2, 9)	9 (6, 22)	<0.001*	6 (2, 12)
Medication, *n* (%)				
Statins	0 (0)	17 (35)	<0.001*	17 (12)
Antihypertensive drugs	0 (0)	23 (46)	<0.001*	23 (17)
Calcium burden				
Thoracic aorta, *n* (%)	18 (20)	30 (60)	<0.001*	48 (35)
Thoracic aorta (mm^3^), median (25th, 75th percentile)	0 (0, 0)	1 (0, 5)	<0.001*	0 (0, 1)
Injected dose (MBq), mean ± SD				
^18^F-FDG	306 ± 59	315 ± 65	0.410	309 ± 61
^18^F-NaF	174 ± 39	175 ± 28	0.851	174 ± 35
Circulating time (min), mean ± SD				
^18^F-FDG	181 ± 4	182 ± 5	0.500	181 ± 4
^18^F-NaF	92 ± 4	91 ± 4	0.339	91 ± 4
Radiotracer activity (kBq/mL), mean ± SD^a^				
FDG_MAX_	8.79 ± 1.69	9.30 ± 1.95	0.085	8.97 ± 1.78
NaF_MAX_	3.36 ± 0.61	3.76 ± 0.76	<0.001*	3.50 ± 0.66
PET/CT system (%, ^18^F-FDG/Na^18^F)				
GE Discovery STE	20/25	16/28		19/26
GE Discovery VCT	31/21	20/18		27/20
GE Discovery RX	25/32	32/20		27/27
GE Discovery 690/710	24/22	32/34		27/27

*HbA1c* Glycated haemoglobin, *eGFR* Estimated glomerular filtration rate

*P < 0.05

^a^Activity concentrations adjusted for blood activity, injected dose and PET/CT technology

**Fig. 2:**
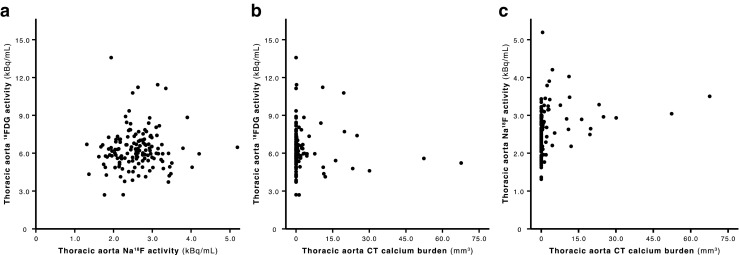
**a** Thoracic aorta ^18^F-FDG activity (FDG_MAX_) versus thoracic aorta Na^18^F activity (NaF_MAX_). FDG_MAX_ is not correlated with NaF_MAX_ (Spearman’s *ρ* = 0.07, *P* = 0.427). **b** FDG_MAX_ versus thoracic aorta CT calcium burden. FDG_MAX_ is not correlated with thoracic aorta CT calcium burden (Spearman’s *ρ* = 0.04, *P* = 0.654). **c** NaF_MAX_ versus thoracic aorta CT calcium burden. NaF_MAX_ is positively correlated with thoracic aorta CT calcium burden (Spearman’s *ρ* = 0.42, *P* < 0.001). (FDG_MAX_ and NaF_MAX_ are the maximum activity concentrations of ^18^F-FDG and Na^18^F, respectively, adjusted for blood activity, injected dose and PET/CT technology)

Thoracic aorta ^18^F-FDG uptake was not correlated with either thoracic aorta Na^18^F uptake or thoracic aorta CT calcium burden, whereas thoracic aorta Na^18^F uptake was positively correlated with thoracic aorta CT calcium burden (Spearman’s *ρ* = 0.42, *P* < 0.001; Fig. 2).

The results of the univariate analysis are presented in Supplementary Tables 2–4. The multivariable linear regression analysis showed that independent determinants of thoracic aorta ^18^F-FDG uptake were subject age (0.41 kBq/mL per SD, *P* = 0.008) and family history (0.66 kBq/mL, *P* = 0.035), which explained an additional 4 % of the variation in thoracic aorta FDG_MAX_ (adjusted *R* ^2^ increased from 0.60 to 0.64, *P* = 0.001). Blood ^18^F-FDG activity, injected ^18^F-FDG dose and PET/CT technology explained the initial 60 % of the variation in the data. The multivariable linear regression analysis showed that independent determinants of thoracic aorta Na^18^F uptake were subject age (0.25 kBq/mL per SD, *P* < 0.001), body mass index (0.12 kBq/mL per SD, *P* = 0.017), renal function (−0.09 kBq/mL per SD, *P* = 0.022) and thoracic aorta CT calcium burden (0.08 kBq/mL per SD, *P* = 0.002), which explained an additional 9 % of the variation in thoracic aorta NaF_MAX_ (adjusted *R* ^2^ increased from 0.71 to 0.80, *P* < 0.001). Blood Na^18^F activity, injected Na^18^F dose and PET/CT technology explained the initial 71 % of the variation in the data. The multivariable linear regression showed that independent determinants of thoracic aorta CT calcium burden were subject age (1.47 mm^3^ per SD, *P* = 0.013) and antihypertensive treatment (10.17 mm^3^, *P* = 0.030), which explained 28 % of the variation in thoracic aorta mean CT calcium volume (adjusted *R* ^2^ = 0.28, *P* < 0.001).

**Fig. 3:**
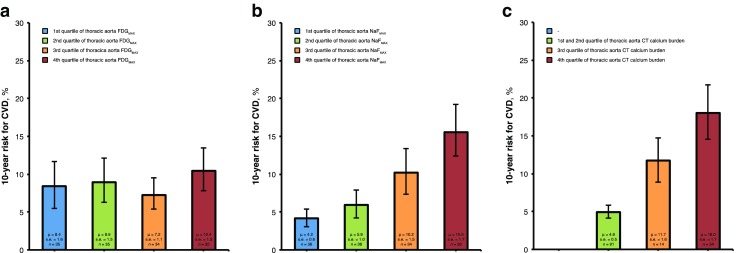
The 10-year cardiovascular disease (CVD) risk estimated by the Framingham risk score in relation to quartiles of (**a**) thoracic aorta ^18^F-FDG activity (FDG_MAX_), (**b**) thoracic aorta Na^18^F activity (NaF_MAX_), and (**c**) thoracic aorta CT calcium burden. CVD risk is similar in all quartiles of thoracic aorta FDG_MAX_, but increases linearly with each increasing quartile of thoracic aorta NaF_MAX_ (*P* < 0.001 for a linear trend) and with each increasing quartile of thoracic aorta CT calcium burden (*P* < 0.001 for a linear trend). *s.e.* standard error, *μ* mean

**Fig. 4:**
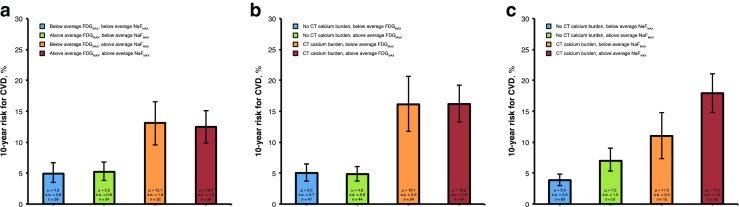
The 10-year cardiovascular disease (CVD) risk, estimated by the Framingham risk score, in (**a**) subjects with below or above average thoracic aorta ^18^F-FDG activity (FDG_MAX_) and Na^18^F activity (NaF_MAX_), (**b**) subjects with or without thoracic aorta CT calcium burden and below or above average FDG_MAX_, (**c**) subjects with or without thoracic aorta CT calcium burden and below or above average NaF_MAX_. NaF_MAX_ and thoracic aorta CT calcium burden differentiated subjects at high and low CVD risk, whereas FDG_MAX_ did not, *μ* mean

FRS was similar in all quartiles of thoracic aorta ^18^F-FDG uptake (*P* = 0.492 for a linear trend, Spearman’s *ρ* = 0.12; *P* = 0.156). In contrast, FRS increased linearly with each increasing quartile of thoracic aorta Na^18^F uptake (*P* < 0.001 for a linear trend, Spearman’s *ρ* = 0.50; *P* < 0.001). FRS was on average 3.7 times higher in subjects with thoracic aorta Na^18^F uptake in the highest quartile compared with those in the lowest quartile of the distribution (15.5 % vs. 4.2 %; *P* < 0.001). FRS also increased linearly in subjects with increasing quartiles of thoracic aorta CT calcium burden (*P* < 0.001 for a linear trend, Spearman’s *ρ* = 0.63; *P* < 0.001). FRS was on average 3.7 times higher among subjects with thoracic aorta CT calcium burden in the highest quartile compared with those in the lowest two quartiles of the distribution (18.0 % vs. 4.9 %; *P* < 0.001; Fig. 3). The multivariable linear regression analysis showed that independent determinants of FRS (adjusted *R* ^2^ = 0.39, *P* < 0.001) were thoracic aorta Na^18^F uptake (*β* = 0.37, *P* < 0.001) and thoracic aorta CT calcium burden (*β* = 0.42, *P* = 0.002), but not thoracic aorta ^18^F-FDG uptake (*β* = 0.10, *P* = 0.113; Table 2, Fig. 4).

**Table 2:** Multivariable linear regression analysis of the dependence of the 10-year cardiovascular disease (CVD) risk, estimated by the Framingham risk score, on thoracic aorta ^18^F-FDG activity (FDG_MAX_), thoracic aorta Na^18^F activity (NaF_MAX_), and the thoracic aorta CT calcium burden (*β* standardized regression coefficient)

Determinant	Regression coefficient (95 % CI)	β	Adjusted *R* ^2^	*P* value
			0.39	<0.001
Intercept (%)	−9.46 (−16.70 to −3.75)			0.007
FDG_MAX_ (kBq/mL)	0.50 (−0.08 to 1.15)	0.10		0.113
NaF_MAX_ (kBq/mL)	5.37 (3.34 to 7.93)	0.37		<0.001
CT calcium burden (mm^3^)	0.41 (0.26 to 1.07)	0.42		0.002

The agreement between mean calcium burden calculated on CT images from ^18^F-FDG PET/CT and those calculated from Na^18^F PET/CT was considered excellent, as indicated by a small interscan difference (Supplementary Figure 1). The results of our study were similar when aortic tracer uptake was expressed as FDG-TBR_MAX/MEAN_ and NaF-TBR_MAX/MEAN_ (Supplementary Fig. 2, Supplementary Tables 5–7).

## Discussion

In this study, an increased risk of CVD, as estimated by the FRS, was found to be associated with marked increases in vascular calcification metabolism, as assessed by Na^18^F PET/CT imaging, and vascular calcium burden, as assessed by CT imaging, but was not associated with arterial inflammation, as assessed by ^18^F-FDG PET/CT imaging. These findings support the use of arterial Na^18^F PET/CT imaging for identifying the vulnerable patient, but the value of arterial ^18^F-FDG PET/CT imaging is less clear.

Arterial inflammation and vascular calcification are regarded as key processes in the pathogenesis of various CVDs, in particular atherosclerosis [4, 5]. Atherosclerotic plaque inflammation is characterized by accumulation of macrophages, which are attracted to plaques in response to the retention of lipids in the arterial intima [18]. Plaque macrophages secrete chemokines, proinflammatory cytokines and matrix metalloproteinases, which contribute to a nonresolving inflammatory response that leads to plaque hypoxia, plaque necrosis, weakening of the protective fibrous cap and, ultimately, plaque rupture [18, 19]. Plaque rupture is considered the most frequent cause of adverse cardiovascular events, such as acute coronary syndromes and stroke [19, 20]. In response to chronic inflammation and necrosis, atherosclerotic plaques calcify [21]. It is believed that atherosclerotic plaque calcification retards the inflammatory response, stabilizes the atherosclerotic plaque, and reduces the risk of plaque rupture. Nonetheless, the earliest stages of plaque calcification are associated with increased plaque instability and an elevated risk of rupture [22], whereas only advanced stages of plaque calcification are associated with plaque stabilization [23]. A possible explanation for the increased risk of plaque rupture during early stages of plaque calcification is that plaque microcalcifications increase local tissue stress, which facilitate plaque vulnerability [24]. Although plaque calcification is associated with plaque stabilization, the presence and degree of vascular macrocalcifications is strongly predictive of adverse cardiovascular events [7]. It appears that vascular calcification, independent of its association with plaque stability, is a marker of overall atherosclerotic disease burden, and thus, the vulnerable patient [3, 25].

By inference, imaging techniques aimed at visualizing arterial inflammation and vascular calcification are potent markers of CVD risk. ^18^F-FDG PET/CT imaging can noninvasively assess arterial inflammation, whereas Na^18^F PET/CT and CT imaging can noninvasively assess vascular calcification. ^18^F-FDG uptake reflects the rate of glycolysis, which is particularly increased in atherosclerotic plaques that retain macrophages [26] and plaques that undergo hypoxic stress [27]. In addition to atherosclerotic plaque, aortic ^18^F-FDG retention has been linked to formation of aneurysms and dissection of the aorta, which are diseases associated with inflammation of the arterial media and arterial adventitia [28]. It has been reported that arterial ^18^F-FDG uptake increases in proportion to CVD risk factors [29] and that aortic ^18^F-FDG retention predicts adverse cardiovascular events beyond traditional CVD risk factors [6]. Na^18^F uptake reflects the active exchange of hydroxyl groups of hydroxyapatite crystals for fluoride producing fluorapatite [30]. This process is believed to reflect calcification metabolism of osseous tissue, including calcification of atherosclerotic plaque [31–33]. While calcification of atherosclerotic plaque, which is regarded as a disease of the arterial intima, arterial Na^18^F retention is believed to also reflect calcification of the arterial media, a condition associated with arterial stiffening, increased pulse pressure, left ventricular hypertrophy, and reduced myocardial perfusion [34, 35]. Similar to arterial ^18^F-FDG retention, arterial Na^18^F retention has also been reported to increase in proportion to CVD risk factors [31, 32]. CT imaging targets structural vascular calcifications. Numerous follow-up studies have shown that the vascular calcium burden, as detected by CT imaging, is a strong independent marker of CVD risk [7, 8].

This study confirmed the findings from previous investigations. First, this study confirmed that arterial ^18^F-FDG retention is not correlated with either arterial Na^18^F retention [10] or CT calcium burden [9]. A previous study has shown that ^18^F-FDG-avid plaques rarely (∼7 %) accumulate Na^18^F and only occasionally (∼15 %) colocate with structural calcium deposits [9]. These findings suggest that arterial retention of ^18^F-FDG and Na^18^F represent different stages of the cardiovascular atherosclerotic disease process and their imaging may help differentiate early from advanced stages of the disease [9] or may carry independent prognostic value. Second, this study confirmed that arterial Na^18^F retention is positively correlated with CT calcium burden [9]. A previous study has shown that structural calcium deposits are present in approximately 77 % of Na^18^F-avid plaques, whereas only 21 % of vascular macrocalcifications retain Na^18^F [9]. These findings suggest that arterial Na^18^F retention may discriminate active from indolent vascular calcifications, associated with vulnerable and stabilized plaques, respectively [10]. Our data seem to confirm this suggestion.  In those with CT calcium burden, but below average aortic Na^18^F uptake, CVD risk was substantially lower compared to those with CT calcium burden and above average aortic Na^18^F uptake (10.5 % vs. 17.5 %). Lastly, this study confirmed that CVD risk, as estimated by the FRS, increases linearly with increasing arterial Na^18^F uptake and CT calcium burden [10, 36].

Despite confirming several findings from previous investigations, our observations challenge the notion that arterial inflammation, as assessed by ^18^F-FDG PET/CT, is associated with an elevated risk of CVD [6]. There may be several explanations for this discrepant finding. First, this study evaluated subjects at low CVD risk (i.e. median FRS of 6 %), whereas the majority of studies investigating arterial ^18^F-FDG retention in relation to CVD risk evaluated subjects at high CVD risk [6]. Therefore, our study might have detected early stages of atherosclerotic plaque inflammation, but not later stages of inflammation that are associated with plaque and patient vulnerability. Second, this study included subjects with a broad age range, that is between 21 and 75 years. Therefore, it can be assumed that subjects with a spectrum of atherosclerosis severity, ranging from mild to severe disease were included. Both fatty streaks (the hallmark of mild atherosclerosis) and high-risk vulnerable plaques (the hallmark of severe atherosclerosis) are densely populated by macrophages [18]. Because ^18^F-FDG is primarily retained in plaque macrophages [37–39], differentiation of fatty streaks from high-risk vulnerable plaques, and thus differentiation of mild from severe atherosclerosis, may be difficult by ^18^F-FDG PET/CT. Third, this study estimated CVD risk solely based on FRS. A previous study has shown that aortic ^18^F-FDG retention predicts CVD risk beyond FRS [6], and also demonstrated that adding the aortic ^18^F-FDG retention index to FRS results in a net reclassification improvement of approximately 25 % compared with FRS alone [6]. Because this study estimated CVD risk solely via FRS, the incremental value of arterial ^18^F-FDG retention over FRS in predicting CVD risk could not be assessed. The three considerations mentioned above may explain the lack of association between arterial ^18^F-FDG retention and CVD risk found in this study.

### Strengths and limitations

An important strength of the present study is that we prospectively investigated the relationship between CVD risk and arterial inflammation, vascular calcification metabolism, and vascular calcium burden in a heterogeneous group of subjects at low CVD risk. Previous studies that have investigated similar relationships either were performed retrospectively in oncology patients [9] or involved exclusively elderly patients with advanced CVD [10]. Such studies may be limited by imaging protocols not necessarily optimized for imaging arteries, by selection bias, or by both. In contrast, this study was performed prospectively, included a heterogeneous group of subjects at low CVD risk, and utilized imaging protocols optimized for artery imaging [13–15]. Thus we were able to demonstrate that CVD risk is positively associated with increases in thoracic aorta Na^18^F uptake and thoracic aortic CT calcium burden, but is not associated with thoracic aorta ^18^F-FDG uptake.

The findings of this study, however, should be interpreted in light of three limitations. First, CVD risk was estimated in terms of the FRS. Although the revised FRS performs well in terms of discrimination and calibration [12], it still tends to overestimate risk in those at low CVD risk and underestimates risk in those at high CVD risk. Our estimates of CVD risk may therefore be inaccurate and there may be bias in the associations between CVD risk and our imaging findings. However, because of the cross-sectional nature of our study, we relied on an estimated CVD risk. Second, the relationships between CVD risk and arterial inflammation, vascular calcification metabolism, and vascular calcium burden were evaluated in a cross-sectional study. A previous study involving serial ^18^F-FDG PET/CT examinations demonstrated that baseline arterial ^18^F-FDG uptake predicts the finding of vascular calcium deposits on the follow-up examination, suggesting a temporal relationship between arterial inflammation and vascular calcification [40]. Temporal relationships are difficult to assess in cross-sectional studies and a longitudinal approach is preferred for such an evaluation. Therefore, our finding that arterial inflammation is not related either to vascular calcification metabolism or to vascular calcium burden could be attributed to our cross-sectional design. Third, ethical considerations prevented collection of arterial specimens for histological examination. Therefore, we could not relate our imaging findings to the exact structure, biological composition and inflammatory state of the detected atherosclerotic plaques [9]. Substantiating arterial ^18^F-FDG and Na^18^F uptake by histopathology, preferably in the early stages of the disease, might have contributed to a better understanding of the metabolic pathways that govern CVD risk.

### Conclusion

Our findings indicate that an unfavourable cardiovascular risk profile is associated with marked increases in thoracic aorta vascular calcification metabolism and calcium burden, but not arterial inflammation. Our findings support the use of arterial Na^18^F PET/CT imaging for identifying the vulnerable patient, but the value of arterial ^18^F-FDG PET/CT imaging is less clear. Nonetheless, prospective long-term follow-up studies are required to assess the risk stratification abilities of arterial ^18^F-FDG and Na^18^F PET/CT imaging beyond standard approaches, such as the FRS and CT calcium score.

## Electronic supplementary material

Below is the link to the electronic supplementary material.

ESM 1 (DOCX 247 kb)
