# Supplementary material for: Thoracic aorta calcification but not inflammation is associated with increased cardiovascular disease risk: results of the CAMONA study
Source: Eur J Nucl Med Mol Imaging. 2016 Oct 29;44(2):249–58. doi: 10.1007/s00259-016-3552-9 (PMC5214929; doi:10.1007/s00259-016-3552-9)
Supplement: Supplementary file 1 — (DOCX 247 kb) [file 259_2016_3552_MOESM1_ESM.docx]

# SUPPLEMENTAL FILES

**Title:** Thoracic Aorta Calcification but not Inflammation is Associated With Increased Cardiovascular Disease Risk: Results of the CAMONA Study

**Authors:** Björn A. Blomberg, Pim A. de Jong, Anders Thomassen, Marnix G.E. Lam, Werner Vach, Michael H. Olsen, Willem P.T.M. Mali, Jagat Narula, Abass Alavi, Poul F. Høilund-Carlsen

**Journal:** European Journal of Nuclear Medicine and Molecular Imaging, 2016

**Files:**

Supplementary figure 1

Supplementary figure 2

Supplementary table 1

Supplementary table 2

Supplementary table 3

Supplementary table 4

Supplementary table 5

Supplementary table 6

Supplementary table 7

# SUPPLEMENTARY FIGURES

## Supplementary figure 1

**Supplementary figure 1** – Difference plot depicting the inter-scan agreement of thoracic aorta CT calcium burden. The inter-scan agreement was considered good (mean difference of 0.20 mm^3^; 95 % limits of agreement 1.73 to -2.13).

**Supplementary figure 2**

| A  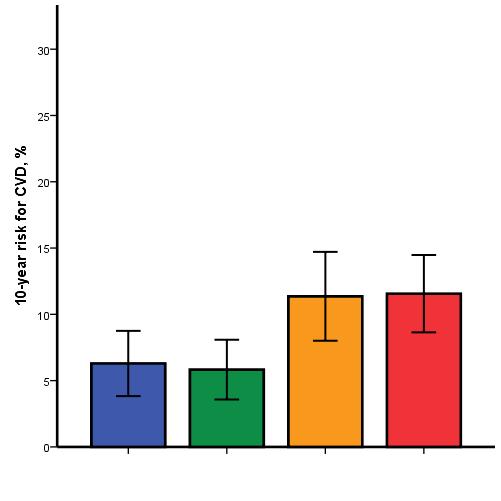 | B  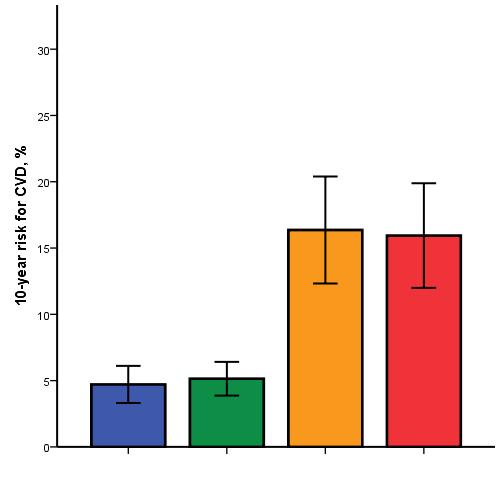 | C  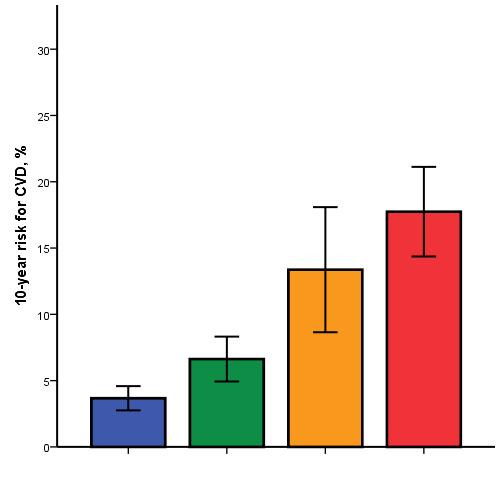 |
| --- | --- | --- |

**Supplementary figure 2** – Bar graph showing the 10-year cardiovascular disease (CVD) risk, estimated by the Framingham Risk Score, against groups below or above average thoracic aorta ^18^FDG activity (FDG-TBR_MAX/MEAN_) and below or above average thoracic aorta Na^18^F activity (NaF-TBR_MAX/MEAN_) (**A**), groups with or without thoracic aorta CT calcium burden and below or above average FDG-TBR_MAX/MEAN_ (**B**), groups with or without thoracic aorta CT calcium burden and below or above average NaF-TBR_MAX/MEAN_ (**C**). NaF-TBR_MAX/MEAN_ and thoracic aorta CT calcium burden differentiated subjects at high CVD risk from subjects at low CVD risk, whereas FDG-TBR_MAX/MEAN_ did not. Abbreviations as in Table 6.

*Blue bar* in A – Below average FDG-TBR_MAX/MEAN_, below average NaF-TBR_MAX/MEAN_

*Green bar* in A – Above average FDG-TBR_MAX/MEAN_, below average NaF-TBR_MAX/MEAN_

*Orange bar* in A – Below average FDG-TBR_MAX/MEAN_, above average NaF-TBR_MAX/MEAN_

*Green bar* in A – Above average FDG-TBR_MAX/MEAN_, above average NaF-TBR_MAX/MEAN_

*Blue bar* in B – No CT calcium burden, below average FDG-TBR_MAX/MEAN_

*Green bar* in B – No CT calcium burden, above average FDG-TBR_MAX/MEAN_

*Orange bar* in B – CT calcium burden, below average FDG-TBR_MAX/MEAN_

*Green bar* in B – CT calcium burden, above average FDG-TBR_MAX/MEAN_

*Blue bar* in C – No CT calcium burden, below average NaF-TBR_MAX/MEAN_

*Green bar* in C – No CT calcium burden, above average NaF-TBR_MAX/MEAN_

*Orange bar* in C – CT calcium burden, below average NaF-TBR_MAX/MEAN_

*Green bar* in C – CT calcium burden, above average NaF-TBR_MAX/MEAN_

# SUPPLEMENTARY TABLES

## Supplementary table 1

| TABLE 1 – **PET/CT system specifications and image reconstruction parameters** | | | | | | | |
| --- | --- | --- | --- | --- | --- | --- | --- |
|  | **System specifications** | |  | **Image reconstruction parameters** | | | |
| **Vendor and type** | **PET scintillator** | **CT** |  | **Iterations/ subsets** | **Post-hoc filter** | **Reconstruction matrix** | **ToF/PSF** |
| GE Discovery STE **[1]** | BGO | 16-slice |  | 2/28 | 6 mm | 128x128 | No/No |
| GE Discovery VCT **[1]** | BGO | 64-slice |  | 2/28 | 6 mm | 128x128 | No/No |
| GE Discovery RX **[2]** | LYSO | 16-slice |  | 2/21 | 6 mm | 128x128 | No/No |
| GE Discovery 690/710 **[3]** | LYSO | 64-slice |  | 3/24 | 5 mm | 256x256 | Yes/Yes |

**SUPPLEMENTARY Table 1** – Summary of PET/CT system specifications and image reconstruction parameters. BGO, LYSO, and ToF/PSF indicate bismuth germanate, lutetium yttrium oxyorthosilicate, and time-of-flight and/or point spread function image reconstruction.

1. Teräs M, Tolvanen T, Johansson JJ et al. Performance of the new generation of whole-body PET/CT scanners: Discovery STE and Discovery VCT. Eur J Nucl Med Mol Imaging. 2007;34:1683-92.

2. Kemp BJ, Kim C, Williams JJ, et al. NEMA NU 2-2001 performance measurements of an LYSO-based PET/CT system in 2D and 3D acquisition modes. J Nucl Med. 2006;47:1960-7.

3. Bettinardi V, Presotto L, Rapisarda E, et al. Physical performance of the new hybrid PET∕CT Discovery-690. Med Phys. 2011;38:5394-411.

## Supplementary table 2

| TABLE 2 – **Determinants of thoracic aorta ^18^FDG uptake** | | | |
| --- | --- | --- | --- |
| **Determinant** | **Crude †** | **Age adjusted** | **Age and sex adjusted** |
| **Sex**, male | -0.21 (-0.87 to 0.48) |  |  |
| **Age**, years | 0.45 (0.18 to 0.72) |  | 0.46 (0.18 to 0.76) |
| **Smoking**, former or current | -0.08 (-0.68 to 0.58) |  |  |
| **Positive Family history**, yes | 0.78 (0.22 to 1.40) | 0.68 (0.07 to 1.34) | 0.66 (0.03 to 1.29) |
| **Blood pressure**, mmHg |  |  |  |
| - Systolic | 0.04 (-0.25 to 0.35) |  |  |
| - Diastolic | 0.12 (-0.16 to 0.40) |  |  |
| **Body mass index**, kg/m^2^ | 0.07 (-0.29 to 0.44) |  |  |
| **Cholesterol**, mmol/L |  |  |  |
| - Total | 0.18 (-0.03 to 0.39) |  |  |
| - LDL | 0.04 (-0.25 to 0.31) |  |  |
| - HDL | 0.27 (-0.09 to 0.68) |  |  |
| **Triglycerides**, mmol/L | 0.05 (-0.27 to 0.38) |  |  |
| **Plasma glucose**, mmol/L | -0.09 (-0.36 to 0.22) |  |  |
| **HbA1c**, mmol/mol | 0.22 (-0.03 to 0.70) |  |  |
| **eGFR**, mL/min/1.73 m^2^ | -0.35 (-0.68 to -0.01) | -0.35 (-0.71 to 0.01) |  |
| **Medication**, yes |  |  |  |
| - Statines | 0.67 (-0.29 to 1.75) |  |  |
| - Antihypertensive drugs | 0.30 (-0.71 to 1.38) |  |  |
| **Vascular calcification**, *n* | 0.10 (-0.70 to 0.89) |  |  |
| **Vascular calcification**, mm^3^ | -0.06 (-0.36 to 0.63) |  |  |
| **Thoracic aorta radioactivity**, kBq/mL |  |  |  |
| - [^18^F]-sodium fluoride | 0.13 (-0.29 to 0.54) |  |  |

**Table 2** – Determinants of thoracic aorta ^18^FDG uptake (FDG_MAX_). Values are expressed as linear regression coefficients (95 % confidence interval) per standard deviation of change in the determinant. To illustrate, a regression coefficient of 0.45 for age signifies that an increase in age with 1 standard deviation (i.e. 14 years) relates to an increase in FDG_MAX_ of 0.45 kBq/mL. † Crude values of FDG_MAX_ were adjusted for blood ^18^FDG activity, injected ^18^FDG dose, and PET/CT technology. Abbreviations as in Table 1.

## Supplementary table 3

| TABLE 3 – **Determinants of thoracic aorta Na^18^F uptake** | | | |
| --- | --- | --- | --- |
| **Determinant** | **Crude †** | **Age adjusted** | **Age and sex adjusted** |
| **Sex**, male | 0.17 (-0.06 to 0.36) |  |  |
| **Age**, years | 0.33 (0.25 to 0.40) |  | 0.32 (0.25 to 0.40) |
| **Smoking**, former or current | 0.17 (-0.02 to 0.34) |  |  |
| **Positive family history**, yes | 0.08 (-0.16 to 0.31) |  |  |
| **Blood pressure**, mmHg |  |  |  |
| - Systolic | 0.09 (-0.01 to 0.19) |  |  |
| - Diastolic | 0.09 (-0.02 to 0.18) |  |  |
| **Body mass index**, kg/m^2^ | 0.15 (0.02 to 0.26) | 0.13 (0.02 to 0.21) | 0.13 (0.03 to 0.21) |
| **Cholesterol**, mmol/L |  |  |  |
| - Total | 0.14 (0.05 to 0.21) | 0.04 (-0.05 to 0.13) |  |
| - LDL | 0.11 (0.02 to 0.18) | 0.04 (-0.04 to 0.11) |  |
| - HDL | -0.07 (-0.17 to 0.05) |  |  |
| **Triglycerides**, mmol/L | 0.20 (0.09 to 0.33) | 0.15 (0.05 to 0.28) | 0.15 (0.05 to 0.29) |
| **Plasma glucose**, mmol/L | 0.16 (0.05 to 0.32) | 0.06 (-0.06 to 0.18) |  |
| **HbA1c**, mmol/mol | 0.18 (0.07 to 0.34) | 0.07 (-0.03 to 0.17) |  |
| **eGFR**, mL/min/1.73 m^2^ | -0.20 (-0.29 to -0.09) | -0.09 (-0.17 to -0.01) | -0.09 (-0.18 to -0.01) |
| **Medication**, yes |  |  |  |
| - Statines | 0.24 (-0.03 to 0.51) |  |  |
| - Antihypertensive drugs | 0.48 (0.19 to 0.75) | 0.18 (-0.08 to 0.45) |  |
| **Vascular calcification**, *n* | 0.55 (0.35 to 0.74) | 0.20 (-0.05 to 0.47) |  |
| **Vascular calcification**, mm^3^ | 0.16 (0.09 to 0.38) | 0.07 (0.01 to 0.14) | 0.06 (0.01 to 0.13) |
| **Thoracic aorta radioactivity**, kBq/mL |  |  |  |
| - [^18^F]-fluorodeoxyglucose | 0.04 (-0.07 to 0.16) |  |  |

**Table 3** – Determinants of thoracic aorta Na^18^F uptake (NaF_MAX_). Values are expressed as linear regression coefficients (95 % confidence interval) per standard deviation of change in the determinant. To illustrate, a regression coefficient of 0.33 for age signifies that an increase in age with 1 standard deviation (i.e. 14 years) relates to an increase in NaF_MAX_ of 0.33 kBq/mL. † Crude values of NaF_MAX_ were adjusted for blood Na^18^F activity, injected Na^18^F dose, and PET/CT technology. Abbreviations as in Table 1.

## Supplementary table 4

| TABLE 4 – **Determinants of thoracic aorta CT calcium burden** | | | |
| --- | --- | --- | --- |
| **Determinant** | **Crude** | **Age adjusted** | **Age and sex adjusted** |
| **Sex**, male | 1.97 (-0.31 to 4.61) |  |  |
| **Age**, years | 3.15 (1.75 to 4.88) |  | 3.30 (1.70 to 5.16) |
| **Smoking**, former or current | 2.90 (0.02 to 5.75) | 1.62 (-1.92 to 4.52) |  |
| **Positive Family history**, yes | -2.04 (-4.13 to -0.14) | -2.60 (-5.29 to -0.56) | -2.32 (-4.67 to -0.56) |
| **Blood pressure**, mmHg |  |  |  |
| - Systolic | 1.26 (0.31 to 2.54) | 0.33 (-0.88 to 1.59) |  |
| - Diastolic | 0.44 (-0.57 to 1.36) |  |  |
| **Body mass index**, kg/m^2^ | -0.10 (-1.29 to 0.99) |  |  |
| **Cholesterol**, mmol/L |  |  |  |
| - Total | 0.47 (-0.54 to 1.43) |  |  |
| - LDL | 0.17 (-1.18 to 1.44) |  |  |
| - HDL | -0.09 (-0.84 to 0.84) |  |  |
| **Triglycerides**, mmol/L | 0.94 (-0.46 to 2.67) |  |  |
| **Plasma glucose**, mmol/L | 3.17 (0.47 to 5.27) | 2.35 (-0.51 to 4.54) |  |
| **HbA1c**, mmol/mol | 3.57 (1.08 to 5.28) | 2.70 (-0.03 to 4.71) |  |
| **eGFR**, mL/min/1.73 m^2^ | -0.86 (-2.99 to 1.08) |  |  |
| **Medication**, yes |  |  |  |
| - Statines | 8.18 (2.12 to 15.39) | 5.16 (-1.70 to 12.80) |  |
| - Antihypertensive drugs | 12.00 (5.86 to 19.37) | 10.17 (4.06 to 17.33) | 9.85 (4.55 to 16.53) |
| **Thoracic aorta radioactivity**, kBq/mL |  |  |  |
| - [^18^F]-fluorodeoxyglucose | -0.31 (-1.93 to 1.31) |  |  |
| - [^18^F]-sodium fluoride | 2.53 (0.97 to 4.91) | 1.33 (0.05 to 3.29) | 1.21 (0.00 to 3.15) |

**Table 4** – Determinants of thoracic aorta CT calcium burden (mm^3^). Values are expressed as linear regression coefficients (95 % confidence interval) per standard deviation of change in the determinant. To illustrate, a regression coefficient of 3.15 for age signifies that an increase in age with 1 standard deviation (i.e. 14 years) relates to an increase in CT calcium burden of 3.15 mm^3^. Abbreviations as in Table 1.

**Supplementary table 5**

| TABLE 5 – **Correlation between thoracic aorta FDG-TBR_MAX/MEAN_, NaF-TBR_MAX/MEAN_ and CT calcium burden** | | | |
| --- | --- | --- | --- |
|  | **Spearman’s *ρ*** | **95 % CI** | ***P*-value** |
| FDG-TBR_MAX/MEAN_ *vs.* NaF-TBR_MAX/MEAN_ | 0.00 | -0.17 – 0.18 | .986 |
| FDG-TBR_MAX/MEAN_ *vs.* Calcium burden, mm^3^ | -0.09 | -0.27 – 0.10 | .320 |
| NaF-TBR_MAX/MEAN_ *vs.* Calcium burden, mm^3^ | 0.18 | 0.00 – 0.33 | **.037** |

**Table 5** – Correlation between thoracic aorta ^18^FDG activity (FDG-TBR_MAX/MEAN_), thoracic aorta Na^18^F activity (NaF-TBR_MAX/MEAN_) and thoracic aorta CT calcium burden. Only NaF-TBR_MAX/MEAN_ demonstrated a positive correlation with the thoracic aorta calcium burden. FDG-TBR_MAX/MEAN_ and NaF-TBR_MAX/MEAN_ indicate the ratio between the maximum thoracic aorta [^18^F]-fluorodeoxyglucose activity concentration (kBq/mL) divided by the mean [^18^F]-fluorodeoxyglucose blood activity concentration (kBq/mL) and the maximum thoracic aorta [^18^F]-sodium fluoride activity concentration (kBq/mL) divided by the mean [^18^F]-sodium fluoride blood activity concentration (kBq/mL).

**Supplementary table 6**

| TABLE 6 – **Determinants of the 10-year risk for CVD** | | | | |
| --- | --- | --- | --- | --- |
| **Determinant** | **Regression coefficient** | **β** | **Adjusted *R*^2^** | ***P* value** |
|  |  |  | .31 | **< .001** |
| Intercept, % | -0.66 (-4.92 to -5.83) |  |  | .836 |
| FDG-TBR_MAX/MEAN_ | -0.03 (-1.68 to 1.63) | 0.00 |  | .974 |
| NaF-TBR_MAX/MEAN_ | 2.59 (0.71 to 4.42) | 0.26 |  | **.005** |
| Thoracic aorta CT calcium burden, mm^3^ | 0.44 (0.25 to 1.16) | 0.46 |  | **.005** |

**Table 2** – Multivariable linear regression assessing the dependence of the 10-year cardiovascular disease (CVD) risk, estimated by the Framingham Risk Score, on thoracic aorta ^18^FDG activity (FDG-TBR_MAX/MEAN_), thoracic aorta Na^18^F activity (NaF-TBR_MAX/MEAN_), and the thoracic aorta CT calcium burden. β = standardized regression coefficient. The 95 % confidence interval is presented in parentheses. Abbreviations as in Table 5.

**Supplementary table 7**

| TABLE 7 – **Relationship between the 10-year cardiovascular disease risk (%) and quartiles of thoracic aorta FDG-TBR_MAX/MEAN_, NaF-TBR_MAX/MEAN_ and CT calcium burden** | | | |
| --- | --- | --- | --- |
|  | ***n*** | **Mean CVD risk** | **95 % CI** |
| **1. FDG-TBR_MAX/MEAN_** |  |  |  |
| – First quartile | 33 | 11.45 | 7.72 – 15.18 |
| – Second quartile | 36 | 6.49 | 4.37 – 8.62 |
| – Third quartile | 35 | 6.76 | 4.37 – 9.15 |
| – Fourth quartile | 35 | 10.63 | 7.35 – 10.17 |
| **2. NaF-TBR_MAX/MEAN_** |  |  |  |
| – First quartile | 34 | 6.36 | 3.73 – 8.93 |
| – Second quartile | 35 | 5.80 | 3.70 – 8.90 |
| – Third quartile | 35 | 10.77 | 7.83 – 13.71 |
| – Fourth quartile | 35 | 12.16 | 8.84 – 15.48 |
| **3. CT calcium burden**, mm^3^ |  |  |  |
| – First quartile | - | - | - |
| – Second quartile | 91 | 4.9 | 4.01 – 5.86 |
| – Third quartile | 14 | 11.7 | 8.26 – 15.23 |
| – Fourth quartile | 34 | 18.0 | 14.56 – 21.51 |

**Table 7** – Relationship between the 10-year cardiovascular disease (CVD) risk, estimated by the Framingham Risk Score, against quartiles of thoracic aorta ^18^FDG activity (FDG-TBR_MAX/MEAN_), quartiles of thoracic aorta Na^18^F activity (NaF-TBR_MAX/MEAN_) and quartiles of thoracic aorta CT calcium burden (mm^3^). No correlation existed between CVD risk and FDG-TBR_MAX/MEAN_ (Spearman’s *ρ* = 0.01; *P* = 0.945), whereas both NaF-TBR_MAX/MEAN_ (Spearman’s *ρ* = 0.35; *P* = < .001 as the CT calcium burden (Spearman’s *ρ* = 0.63; *P* < .001) were positively associated with CVD risk.
